# Behavioral Intention to Get a Booster Dose of COVID-19 Vaccine among Chinese Factory Workers

**DOI:** 10.3390/ijerph19095245

**Published:** 2022-04-26

**Authors:** Kechun Zhang, Yuan Fang, Paul Shing-fong Chan, He Cao, Hongbiao Chen, Tian Hu, Yaqi Chen, Xiaofeng Zhou, Zixin Wang

**Affiliations:** 1 Longhua District Center for Disease Control and Prevention, Shenzhen 518110, China; zkc1317@yeah.net (K.Z.); caohe0312@163.com (H.C.); gesila2021@163.com (H.C.); ht1137571641@126.com (T.H.); chloe4697@163.com (Y.C.); zxf20220312@163.com (X.Z.); 2 Department of Health and Physical Education, The Education University of Hong Kong, Hong Kong 999077, China; lunajoef@gmail.com; 3 JC School of Public Health and Primary Care, Faculty of Medicine, The Chinese University of Hong Kong, Hong Kong 999077, China; pchan@link.cuhk.edu.hk

**Keywords:** booster dose, COVID-19 vaccine, behavioral intention, perceptions, information exposure, factory workers, China

## Abstract

China started to offer a booster dose of COVID-19 vaccine to members of the adult population in October 2021. This study investigated the behavioral intention to receive a booster dose of COVID-19 vaccine among factory workers who had completed their primary vaccination series. Participants were full-time factory employees at least 18 years of age in Shenzhen, China. In Shenzhen, factory workers need to receive a physical examination every year. The study sites covered all six organizations providing physical examinations for factory workers. All eligible workers attending these sites between 26 and 31 October 2021 were invited to complete an online survey. This study was based on 2329 participants who had completed the primary COVID-19 vaccination series. Two-level logistic regression models were fitted. Among the participants, 84% intended to receive a free booster dose of COVID-19 vaccine within the next six months. After controlling for significant background characteristics, we found that perceptions related to a booster dose as well as interpersonal level factors such as information exposure on social media, thoughtful consideration of the veracity of the information, and satisfaction with vaccine-related promotional materials were determinants of behavioral intention. Factory workers in China reported a high level of behavioral intention to receive a booster dose of the COVID-19 vaccine.

## 1. Introduction

Vaccination and other behavioral preventive measures can help eradicate the ongoing Coronavirus Disease 2019 (COVID-19) pandemic [1]. As of November 2021, over half of the world’s population had received at least one dose of a COVID-19 vaccine [2]. However, there were concerns that the vaccine-elicited antibody level would drop over time after people completed their primary COVID-19 vaccination series [3,4,5]. The outbreak of Delta and Omicron variants in countries/regions where COVID-19 vaccination program was first rolled out indicated a high degree of waning immunity to the vaccines [6].

A booster dose refers to COVID-19 vaccines administered to people who have completed a primary vaccination series [7]. Existing evidence consistently showed that receiving a booster dose of mRNA, adenovirus vector, or inactivated COVID-19 vaccine could significantly increase antibody titers, including neutralizing antibodies against wild-type virus and variants of concern among healthy adults [4,5,8,9]. Moreover, the booster dose of COVID-19 vaccine was safe and well-tolerated [4,5,8,9,10]. In Israel, a booster dose of BNT162b2 significantly reduced the rate of confirmed infection and serious illness at population level [6]. During the study period, all adults who received inactivated or adenovirus vector vaccines were recommended to receive a booster dose at least six months after completing their primary vaccination series in China [11]. It is suggested that people receive the same vaccine used in their primary vaccination series as a booster dose [11]. The country started to offer booster doses on October 26, 2021. At the time of this study, the coverage of the booster dose remained low worldwide (0.9/100 people), with the highest rate observed in Israel (44.73/100 people) [2]. As of October 26, China administered 2.25 billion doses of COVID-19 vaccines to its 1.4 billion people [12]. However, there was no reporting about the coverage of booster doses in China.

The World Health Organization (WHO) identified vaccine hesitancy as a major threat to global health [13]. People may be hesitant to receive a booster dose of COVID-19 vaccine. Ten studies investigated behavioral intention to receive a booster dose of COVID-19 vaccine [14,15,16,17,18,19,20,21,22,23]. A global online survey showed that 62–96% of adults across countries intended to receive a booster dose if it became available [14]. Another report showed that 26–55% and 11–31% of the general population in the United Kingdom and the United States would take a booster dose without having a COVID-19 test first, respectively [15]. The prevalence of willingness to receive a booster dose among the general population was 62–67% in the United States [16,17], 67.4–71% in Poland [18,19], and 93.7% in China [20]. Regarding healthcare workers, 83.6% in the United States would accept a hypothetical annual booster dose of COVID-19 vaccine [21], and 71.3% in the Czech Republic were willing to accept a booster dose [22]. In Japan, 89.1% of medical students intended to receive a booster dose of COVID-19 vaccine [23]. The general population, healthcare workers, and medical students all shared similar facilitators and barriers to receiving a booster dose of COVID-19 vaccine. Previous COVID-19 vaccination history, perceived high risk of COVID-19, perceived effectiveness of a booster dose against severe illness/symptomatic infection/community transmission, and having a significant other suggesting a booster dose were associated with higher intention to receive a booster dose [14,15,16,17,18,19,20,21,22,23]. Barriers to receiving a booster dose included side effects experienced after primary doses, lack of confidence in the effectiveness and safety of the booster dose, concerns about the sustainability of immunogenicity induced by the booster dose, and lack of trust in the information given by public health/governmental agencies [14,15,16,17,18,19,20,21,22,23]. In addition, concerns about global or national vaccine justice were barriers to receiving a booster dose among Czech healthcare workers [22].

This study targeted factory workers in China. As many factories are crowded and it is difficult for employees to maintain physical distancing, factory workers may have a higher risk of COVID-19 than the general population [24,25]. Many countries have reported COVID-19 outbreaks in workplaces, including China. Therefore, many countries implemented COVID-19 prevention measures for manufacturing industries and mining sites, such as carrying out a COVID-19 risk assessment, developing cleaning and hygiene procedures, helping people work from home, maintaining physical distancing in workplaces, complying with personal preventive measures, and facilitating employees to receive a COVID-19 vaccine [26,27]. These regularities might have an impact on policies related to booster doses of COVID-19 vaccination.

In this study, we covered potential determinants of behavioral intention to receive a booster dose of COVID-19 vaccine at both the individual and interpersonal level under the socioecological model [28]. The socioecological model is commonly used to understand determinants of COVID-19 vaccination in different populations [29,30,31,32,33]. At the individual level, perceptions related to a booster dose may influence a person’s decision to receive it. The Health Belief Model (HBM) was used to guide the selection of variables [33]. Constructs of HBM, such as perceived susceptibility to and severity of COVID-19 and the perceived benefits, barriers, cues to action, and efficacy of the vaccines were determinants of acceptance and/or uptake of COVID-19 vaccination [34,35,36]. Perceptions related to a booster dose based on the HBM influenced people’s decision to receive it [20]. As there are several types of COVID-19 vaccines available as a booster dose, people may experience “choice overload” and find it difficult to select one of them [37]. Decisional conflicts refer to personal uncertainty about which option to take when there are competing options. This study tested whether decisional conflicts in choosing a booster dose could be a barrier.

At the interpersonal level, COVID-19 vaccination is a hot topic on social media [38]. Misinformation related to the COVID-19 vaccines threatens vaccine uptake [38]. Chinese factory workers with a higher frequency of exposure to positive information related to COVID-19 vaccination were more likely to receive a primary COVID-19 vaccination series [29]. It is possible that people exposed to more information supporting a booster dose of COVID-19 vaccine on social media are more likely to accept it. Thoughtful consideration of the veracity of information to which they were exposed was significantly associated with better health outcomes among factory workers in China in the early phase of the COVID-19 outbreak [25]. Such practices may mitigate the negative impact of misinformation related to a booster dose of COVID-19 vaccine as well. People who frequently think about the veracity of information may have higher intention to receive a booster dose. In China, the government is actively disseminating COVID-19 vaccination-related promotional materials through multiple channels. It is important to understand the target audience’s satisfaction with these materials, as there were reports suggesting that promotional materials might not address people’s greatest concerns related to COVID-19 vaccine [39]. Good health-related promotional materials should contain an appropriate amount of information, address the target audience’s greatest concerns, and help them to make decisions. We hypothesized that higher satisfaction with these governmental promotional materials would be associated with higher intention to receive a booster dose of COVID-19 vaccine.

To address existing knowledge gaps, this study investigated behavioral intention to receive a booster dose of COVID-19 vaccination among Chinese factory workers who had completed a primary vaccination series. We examined the effects of the following factors: sociodemographic factors, personal COVID-19 preventive measures, COVID-19 preventive measures implemented by factories, and individual and interpersonal variables.

## 2. Materials and Methods 

### 2.1. Study Design

This manuscript was based on a subsample of 2626 factory workers who completed a cross-sectional online survey in Shenzhen, China conducted between 26 and 31 October 2021. The online survey was the second wave of serial cross-sectional surveillance of COVID-19 vaccination uptake and attitudes among factory workers conducted by the Longhua Center for Disease Control and Prevention (CDC). The study sites, sampling, and data collection method of this study was the same as those conducted during the first wave [29,30]. Shenzhen is a major special economic zone in China, bordering Hong Kong to the south. The majority of the factories here are located in the Longhua district of Shenzhen. By the end of 2020, there were 1517 factories employing one million factory workers in Longhua in 2018 [40]. Three types of COVID-19 vaccines, including two inactivated vaccines (Sinopharm and Sinovac CoronaVac) and one adenovirus vector COVID-19 vaccine (CanSino: Ad5-nCoV), were available in Shenzhen during the study period. 

### 2.2. Participants and Data Collection

The inclusion criteria were that participants be (1) at least 18 years of age and (2) a full-time employee of a factory in Shenzhen. Factory workers in Shenzhen need to take a physical examination every year. Physical examinations are provided by designated public or private hospitals and the CDC. Our study sites for recruitment covered all six organizations providing physical examination services to factory workers in Longhua, including three public hospitals, two private hospitals, and one district CDC. The fieldworkers approached all adults attending these sites for physical examination between 26 and 31 October 2021. Fieldworkers obtained informed consent from all potential study participants.

The Questionnaire Star, an encrypted online survey platform commonly used in China, was used to carry out the survey. Participants scanned Quick Response (QR) codes to access an electronic consent form and online questionnaire on a website. Participants signed the electronic consent form before start filling out the online survey. To avoid duplicate responses, the Questionnaire Star tool only allowed each mobile device to access the online questionnaire once. The participants were asked not to disseminate the QR code to other people. The online survey had four pages with about 20 items per page, and took about 20 min to complete. Before participants submitted the questionnaire, the Questionnaire Star tool performed completeness checks. Participants were able to review and change their responses. Upon completion, an e-coupon for CNY 10 (USD 1.5) was sent to participants as a token of appreciation. All data were stored on the online server of Questionnaire Star and protected by a password. Only the corresponding author had access to the database.

**Figure 1:**
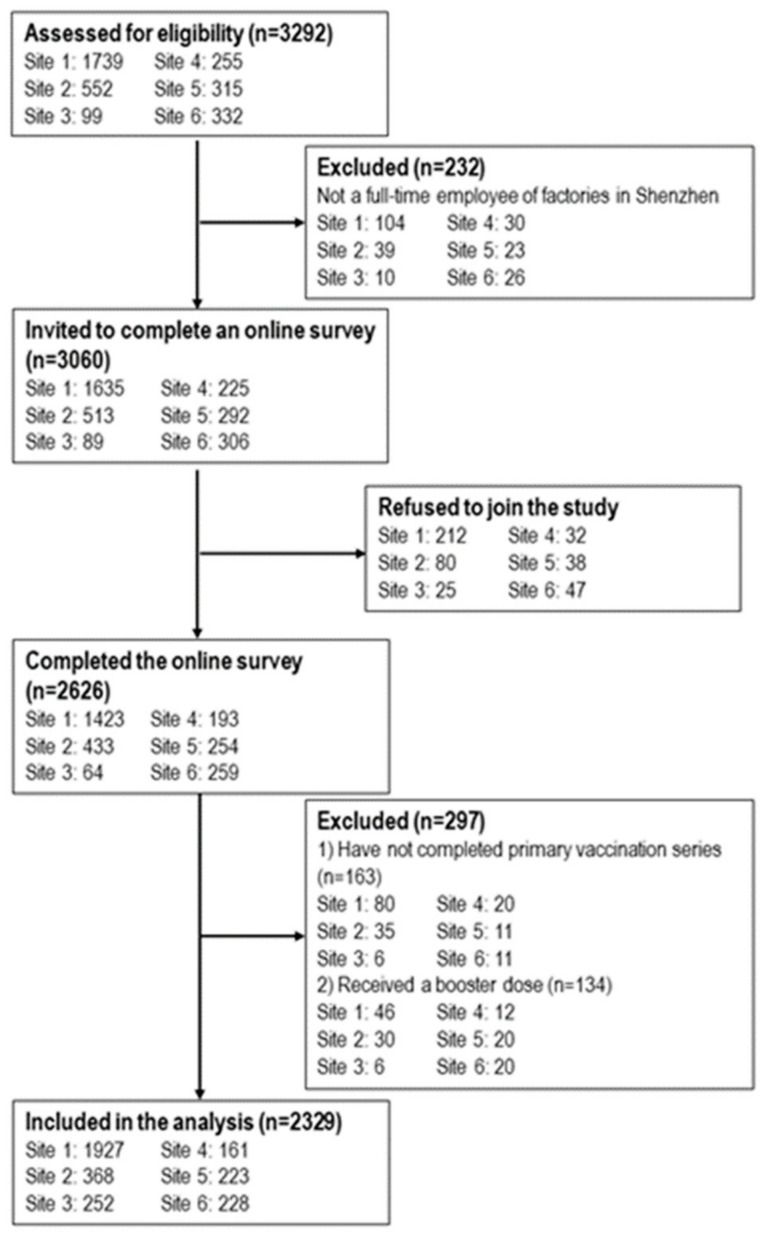
Flowchart of the study.

During the study period the fieldworkers approached 3060 eligible factory workers (between 89 and 1635 across study sites), of whom 2626 completed the online survey (between 64 and 1423 across study sites). The response rate ranged from 71.9% to 87.0% at different sites (overall 85.8%) (Figure 1). Lack of time and other logistical reasons were the main reasons for refusal. Completion of a primary vaccination series was defined as receiving two doses of inactivated COVID-19 vaccine (Sinopharm and/or Sinovac CoronaVac) or one dose of adenovirus vector COVID-19 vaccine (CanSino Ad5-nCoV). At the time of the survey, 163 participants had not completed a primary vaccination series, and 134 had already received a booster dose of COVID-19 vaccine. This study was based on the sub-sample of 2329 participants who had completed a primary vaccination series and had not received a booster dose of COVID-19 vaccine. Ethical approval was obtained from the Longhua District CDC (reference 2021015).

### 2.3. Measurements

#### 2.3.1. Questionnaire Development

One CDC worker, two public health researchers, and a health psychologist formed a panel for questionnaire development. In-depth interviews were conducted to understand factory workers’ attitudes toward a booster dose of COVID-19 vaccine. Purposive sampling was used to recruit adult full-time employees of factories in Shenzhen who had completed their primary COVID-19 vaccination series. Prior to the interview, informants were briefed about the purposes and nature of the interviews. Written informed consent was sought, and clarification prior to the interview was available. The face-to-face interviews were conducted in a quiet place with privacy, and were audio-recorded with the informants’ consent. Mandarin was used as the medium of conversation. Two frontline factory workers (Informant A, male, aged 34 and Informant B, female, aged 26) and one senior factory manager (Informant C, male, aged 46) completed the interviews, which lasted between 1 and 1.5 h. Interviews were transcribed. A code book was kept to record special data and to transform the data into categories to identify main themes. All three informants intended to receive a booster dose in near future. We identified four themes related to facilitators: (1) perceived new variants of COVID-19 as a threat; (2) concern about the protection conferred by the primary vaccination series declining over time; (3) exposure to information on the internet advocating a booster dose; and (4) suggestions from friends and supervisors to receive a booster dose. Three themes were related to barriers: (1) concerns that the side effects of a booster would be stronger than those of the primary doses; (2) concerns about the short duration of protection of a booster dose; and (3) not knowing which type of vaccine was more suitable for them as a booster dose. The panel developed the measurements of the online survey based on these qualitative findings.

The questionnaire was tested for readability and length by another twenty factory workers. All participants in the pilot testing agreed that the wordings and length of the questionnaire were appropriate and easy to understand. The panel then finalized the questionnaire for the actual survey. The twenty participants in the pilot testing did not participate in the actual survey. 

#### 2.3.2. Background Characteristics 

Participants reported their sociodemographic information such as age, gender, internal migrant status, relationship status, number of children, education level, monthly personal income, whether they were frontline workers or management staff, and the type of factory they worked in. Participants reported the type of COVID-19 vaccine they received in the primary vaccination series, the time interval between completion of the primary vaccination series and the survey date, and the severity of side effects they experienced from the primary COVID-19 vaccination series. In addition, participants were asked about their compliance with personal preventive behaviors in the past month, including their frequency of wearing facemasks when having close contact with others in the workplace and other public spaces and sanitizing their hands after returning from public spaces or touching public installations, with possible response categories being every time, often, sometimes, and never. This study measured two physical distancing behaviors, avoiding social/meal gatherings with people who do not live together during the past month and avoiding crowded places during the past month. The same measurements of personal preventive behaviors and physical distancing have been used in previously published studies [24,25,29,30,31,41]. Regarding COVID-19 preventive measures implemented by factories, we added one more item, “requiring employees to receive COVID-19 vaccination”, to the validated eight-item measurement [24,25].

#### 2.3.3. Dependent Variable: Behavioral Intention to Receive a Booster Dose of COVID-19 Vaccine

Participants were briefed with the following statement: “A booster dose of COVID-19 vaccine refers to receiving one more dose of vaccine after completing the primary vaccination series”. Participants were then asked about the likelihood of receiving a free booster dose of COVID-19 vaccine in the next six months (response categories: 1 = very unlikely, 2 = unlikely, 3 = neutral, 4 = likely, 5 = very likely). Behavioral intention was defined as “likely” or “very likely”. The same definition has been commonly used in previous studies [29,30]. Participants with such an intention were further asked about their preference of booster dose (1 = the same type of China-made vaccine as their initial doses, 2 = a China-made vaccine different from their initial doses, 3 = a foreign-developed vaccine, 4 = no preference). 

#### 2.3.4. Independent Variables at the Individual Level

Two single items measured perceived susceptibility (perceived risk of contracting the Delta variant of COVID-19) and perceived severity (perceived chance of having severe illness after contracting the Delta variant of COVID-19) (response categories: 1 = low, 2 = moderate, 3 = high). Two scales were constructed for this study: 1) a three-item Perceived Benefit Scale (e.g., receiving a booster dose can maintain your antibody level and strengthen protection against COVID-19) and 2) a three-item Perceived Barrier Scale (e.g., the perceived likelihood of severe side effects after receiving a booster dose) (response categories: 1 = disagree, 2 = neutral, 3 = agree). The Cronbach’s alpha of these two scales were 0.78 and 0.79; single factors were identified by exploratory factor analysis, explaining 70.1% and 70.5% of the total variance. In addition, cues to action (suggestions from friends/family to receive a booster dose) and perceived self-efficacy (ease of receiving a booster dose of COVID-19 vaccine if desired) were measured by two single items (response categories: 1 = disagree, 2 = neutral, 3 = agree). Decision conflict regarding the choice of a booster dose was measured by the validated Chinese version of the SURE test version of the Decisional Conflict Scale (Cronbach’s alpha of 0.88 in this study) [42]. 

#### 2.3.5. Independent Variables at the Interpersonal Level

We measured frequency of exposure to four different types of information (e.g., COVID-19 pandemic is not under control in some countries after scaling up COVID-19 vaccination) on social media platforms such as WeChat, WeChat moments, Weibo, and Tiktok in the prior month. The responses categories were 1 = almost never, 2 = seldom, 3 = sometimes, and 4 = always. A validated item measured the frequency of thoughtful consideration of the veracity of health information [25]. In addition, three questions assessed satisfaction with COVID-19 vaccination promotional materials produced by the government, including the amount of information provided, whether they addressed concerns related to vaccination, and whether they were helpful in supporting decision-making.

### 2.4. Sample Size Calculation

Assuming the behavioral intention to receive a booster dose of COVID-19 vaccine in the reference group (without a facilitating condition) to be 30–70%, a sample size of 2000 could detect a smallest crude odds ratio (OR) of 1.29 between those with and without such facilitating conditions (power: 0.80; alpha: 0.05; PASS 11.0; NCSS, LLC). Assuming a response rate of 60–70%, around 3000 eligible factory workers were therefore invited to join the study.

### 2.5. Ethics Statement

All subjects provided their informed consent for inclusion before they participated in the study. Ethical approval was obtained from the Longhua District CDC (reference 2021015).

### 2.6. Statistical Analysis

We used the binary variable of behavioral intention to receive a booster dose of COVID-19 vaccine as the dependent variable. Factors associated with the dependent variable were analyzed using two-level logistic regression models (level 1: study sites; level 2: individual participants). We fitted random intercept models to allow the intercept of the regression model to vary across study sites, which could account for intracorrelated nested data. Similar multilevel logistic regression models have been used in other studies with similar sampling methods [29]. The significance of the association between each background characteristic and the dependent variable was first assessed using a univariate two-level logistic regression model. Background characteristics with *p* < 0.05 in the univariate analysis were adjusted in the multivariate two-level logistic regression model. Each multivariate logistic regression model contained one independent variable of interest (individual and interpersonal level factors) and all significant background characteristics. OR, adjusted odds ratios (AOR), and 95% confidence intervals (CI) were obtained. SPSS version 26.0 (IBM Corp) was used for data analysis, with *p* < 0.05 considered statistically significant.

## 3. Results

### 3.1. Background Characteristics of the Participants

The majority of the participants were under 40 years old (72.1%), internal migrants (88.6%), married (71.8%), having a child (69.4%), frontline workers (68.0%), and working in electronic device manufacture (60.1%). About half of them were male (49.2%), did not receive tertiary education (54.0%), and had a monthly personal income below CNY 5000 (USD 773.07) (42.1%). Over 90% of them completed their initial doses within the past six months, and 65.3% did not experience side effects after COVID-19 vaccination. Participants reported good compliance with facemask wearing in both the workplace (73.3%) and other public spaces (85.7%). However, fewer participants had practiced hand hygiene and physical distancing during the past month. About 74% of the participants reported that their factories required employees to take a COVID-19 vaccine (Table 1).

### 3.2. Behavioral Intention to Receive a Booster Dose of COVID-19 Vaccine

Among the participants, 84% had an intention to receive a free booster dose of a COVID-19 vaccine. Among participants with such an intention (*n* = 1956), 84% preferred the same China-made vaccine used in their primary vaccination series, 2.2% preferred a different China-made vaccine, 1.7% preferred vaccines made by foreign countries, and 11.1% did not have a preference (Table 2).

### 3.3. Independent Variables at Individual and Interpersonal Levels

About half of the participants perceived a high risk of contracting the Delta variant of COVID-19 (60%) and a high chance of having severe consequences if contracting it (47.2%). About 70% perceived benefits of receiving a booster dose, while fewer than 20% perceived barriers to receiving a booster dose. The majority of them believed that their significant others would suggest they receive a booster dose (73.3%), while a smaller majority believed that receiving a booster dose would be easy for them (62.4%). The mean score of the SURE test version of the Decisional Conflict Scale was 1.4 (SD: 1.6).

Regarding interpersonal-level variables, about half of the respondents said that they had sometimes/always been exposed to information regarding the COVID-19 pandemic not being under control in other countries which scaled up COVID-19 vaccination (49.0%), the infectiousness and harms of the Delta variant of COVID-19 (61.9%), outbreak of the Delta variant of COVID-19 in China (50.3%), but fewer were exposed to information on people infected with COVID-19 after completing an initial series of vaccination (32.6%). About 27.8% of the participants always considered the veracity of COVID-19-specific information. Most participants were satisfied with the COVID-19 vaccination promotional materials produced by the government (65.9–87.4%). (Table 2)

### 3.4. Factors Associated with Behavioral Intention to Receive a Booster Dose of COVID-19 Vaccine

In the univariate analysis, gender, relationship status, education level, monthly personal income, status as frontline workers or management staff, frequency of wearing a facemask in public spaces/transportation other than the workplace, avoiding social gathering and crowded places, and the number of COVID-19 prevention measures implemented by their factories were all associated with behavioral intention to receive a booster dose of COVID-19 vaccine (Table 3).

After adjusting for significant background characteristics, higher perceived risk (AOR: 1.53, 95% CI: 1.24, 1.90) and higher perceived chance of having severe illness if contracting the Delta variant of COVID-19 (AOR: 1.45, 95% CI: 1.18, 1.77) were associated with higher intention to receive a booster dose. Higher perceived benefit of the booster dose (AOR: 1.71, 95% CI: 1.57, 1.87), agreeing that their significant other would suggest they receive a booster dose (AOR: 2.53, 95% CI: 2.09, 3.77), and perceived higher self-efficacy (AOR: 2.04, 95% CI: 1.72, 2.43) were positively associated with the dependent variable. A negative association was found between perceived barriers and the dependent variable (AOR: 0.80, 95% CI: 0.74, 0.85). At the interpersonal level, higher exposure to information about the COVID-19 pandemic in countries which had scaled up COVID-19 vaccination (AOR: 1.26, 95% CI: 1.13, 1.41), infectiousness and harms of the Delta variant (AOR: 1.38, 95% CI: 1.23, 1.54), and outbreak of the Delta variant of COVID-19 in China (AOR: 1.32, 95% CI: 1.16, 1.49) were associated with higher intention to receive a booster dose. Thoughtful consideration of the veracity of COVID-19-specific information (AOR: 1.35, 95% CI: 1.21, 1.50), belief that governmental health promotional materials could address their concerns related to COVID-19 vaccination (AOR: 2.04, 95% CI: 1.62, 2.57) and were helpful for them in making the decision to receive COVID-19 vaccination (AOR: 3.87, 95% CI: 2.91, 5.14) were positively associated with the dependent variable (Table 4).

## 4. Discussion

The findings of this study represented the latest estimate of acceptance of a booster dose in the early phase of rollout in China, which can be used to project future uptake of a booster dose among factory workers. Factors at the individual and interpersonal levels were determinants of behavioral intention to receive a booster dose of COVID-19 vaccine. This study extended the application of the socioecological model, allowing us to understand the determinants from a comprehensive perspective.

China is in a good position to scale up receipt of a booster dose of COVID-19 vaccine in factory workers. At the time of this study, 93.8% (2463/2626) of the sampled factory workers had completed a primary vaccination series, and about 60% had completed it at least four months prior. A booster dose was highly accepted by factory workers, as 84% of them intended to receive it within the next six months. This level of behavioral intention is comparable to that of the general population in China (93.7%) [20] and to that of healthcare providers in the United States (83.6%) and medical students in Japan [21,23], and is higher than that of the general population in other countries [14,15,16,17,18,19]. However, given the gap between intention and actual behavior [43], effective health promotion is needed to facilitate factory workers in translating this intention into actual behavior.

This study has numerous practical implications for developing health promotion. In contrast to the findings among the general population in the United States and the United Kingdom [15,16], male workers had a lower intention to receive a booster dose of COVID-19 vaccine. More attention should be given to male workers in future programs. Compared to those who were currently single, those with a stable partner had higher intention. Previous studies have shown that being married or cohabiting with a partner was associated with higher COVID-19 vaccine uptake [31]; protecting one’s stable partner might be a motivation to receive a booster dose. Low education and income levels were associated with lower intention to receive a booster dose. Previous studies suggested that factory workers with lower socioeconomic status had lower intention to receive the primary COVID-19 vaccination series [29]. Compared to management staff, frontline workers had lower intention to receive a booster dose. Previous studies had shown that management staff were more likely to adopt COVID-19 preventive measures [24] and receive primary COVID-19 vaccination series [29]. Moreover, higher compliance with facemask wearing in public spaces and physical distancing was associated with higher intention to receive a booster dose. These people may have a stronger motivation to protect themselves against COVID-19, and likely considered receiving a primary COVID-19 vaccination series and booster dose to be a useful means of protection [24,29]. Furthermore, higher number of COVID-19 preventive measures implemented by factories was associated with higher intention to receive a booster dose. Factories might have cultivated widely-shared organization norms favoring COVID-19 vaccination through the implementation of these measures [24,44].

Modifying perceptions related to a booster dose is potentially useful in health promotion, as such promotion was significantly associated with behavioral intention to receive a booster dose. It is necessary to increase the perceived susceptibility and perceived severity of contracting the Delta variant of COVID-19, as these perceptions were facilitators. Health communication messages might emphasize that, as compared to the wild-type virus and other variants of concern (e.g., the Alpha variant), the Delta variant has increased infectiousness and patients infected with the Delta variant have a higher risk of hospitalization and require a longer time for recovery [45,46]. It is useful to enhance the perceived benefit of a booster dose, as this was another facilitator. Evidence of a booster dose in reducing mortality and severe consequences as a result of COVID-19 should be disseminated to factory workers in nonprofessional terms. Building confidence related to the supply of booster doses may be a useful strategy as well. Although only a few participants had concerns about the safety and duration of protection of a booster dose, such concerns need to be reduced, as they were barriers. Testimonials on experiences shared by peers who have received a booster dose might be useful in reducing concerns about side effects. Workers should be updated regularly about the latest evidence on the long-term safety and duration of protection of a booster dose. Cues to action and perceived self-efficacy were both facilitators. Future programs might consider involving the significant others of factory workers in order to provide a strong cue to action to receive a booster dose. Having an outreach team providing a booster dose in factories may be helpful in increasing perceived self-efficacy. In contrast to our hypothesis, decisional conflict regarding the choice of a booster dose was not a barrier. Our findings were that, as expected, health authorities in China provide clear suggestions about the choice of a booster dose. More than 80% of the participants indicated that they would follow such suggestions.

With the onset of the COVID-19 pandemic, social media has rapidly become a crucial communication tool for information generation, dissemination, and consumption [47]. COVID-19 vaccination and the Delta variant are hot topics on social media, as about half of the participants were sometimes/always exposed to these topics on social media within the past month. Higher exposure to these topics was associated with higher intention to receive a booster dose. After knowing that the COVID-19 pandemic was not under control in countries after scaling up COVID-19 vaccination, workers might believe that completing a primary vaccination series would not be sufficient for pandemic control. Understanding more about the infectiousness, harms, and outbreaks caused by the Delta variant might increase their perceived susceptibility to and the perceived severity of this variant. Perceived susceptibility and perceived severity were both facilitators of intention to receive a booster dose in this study. Health authorities should consider using their official social media accounts to disseminate health communication messages promoting a booster dose, as Chinese factory workers considered these official social media accounts to be credible information sources [24]. Our findings highlight the role of thoughtful consideration of the veracity of information specific to COVID-19 in reducing vaccine hesitancy. Thoughtful consideration may mitigate the negative impacts of misinformation on intention to receive COVID-19 vaccination. However, only 30% of the participants always thought carefully about the veracity of information specific to COVID-19. This proportion was slightly lower than that observed in the early phase of the COVID-19 outbreak in the same population [25]; thus, there is a need for improvement. Most of the participants were satisfied with the amount of information in vaccine health-related promotional materials produced by the government, and believed they were helpful in making a decision about receiving a COVID-19 vaccine. However, about one third indicated that these materials did not address their main concerns, and such beliefs were associated with behavioral intention to receive a booster dose. Currently, most public health interventions are developed using a top-down approach where end-users’ involvement is limited, with most intervention components designed and directed by academics and healthcare professionals [48]. These interventions are standardized without considering the needs of end-users from their perspectives [49]. Making use of co-creation, which refers to the collaborative public health intervention development by academics alongside end-users and other non-academic stakeholders [50,51,52] may be helpful in improving these materials. Such an approach is considered to be a promising and efficient solution to addressing complex issues and fostering behavioral change [50,53].

This study has several limitations. First, we did not collect qualitative data from the participants of the online survey. As the survey was anonymous, we were not able to contact the participants and invite them to complete an additional qualitative study. Future studies integrating quantitative and qualitative methods would add breadth and depth of understanding regarding the facilitators of and barriers to intention to receive a booster dose. Second, this study did not study the general population in Shenzhen. We only included factory workers in one Chinese city. Generalizations should therefore be made cautiously. Third, as the study was anonymous and did not collect participants’ identifiable information, we were not able to collect information about those who refused to join the study. Factory workers who refused to join the study might have different characteristics compared to study participants. Selection bias might exist. However, our response rate (85.8%) was higher than another online survey of similar topics [14,15,16,17,18,19,20,21,22,23]. Fourth, data were self-reported and verification was not feasible; thus, recall bias existed. Participants might have over-reported their behavioral intentions due to perceived social desirability. Fifth, measures of perceptions related to a booster dose were self-constructed based on those assessing attitudes toward primary COVID-19 vaccination series among Chinese factory workers [29]. While the internal validity of the self-constructed scales was acceptable, these scales might require external validation as well. Moreover, as this was a cross-sectional study, causal relationships cannot be established.

## 5. Conclusions

Chinese factory workers have a high level of behavioral intention to receive a booster dose of COVID-19 vaccine. Perceptions related to booster doses, information exposure on social media, thoughtful consideration of the veracity of the information, and satisfaction with vaccine-related promotional materials were determinants of behavioral intention. Future programs promoting a booster dose may consider modifying perceptions, such as increasing the perceived susceptibility to and severity of COVID-19 variants of concern, the perceived benefits of a booster dose, and perceived self-efficacy to receive a booster dose. Reducing concerns about safety and the duration of protection may be a useful strategy as well. Health authorities should consider health promotional materials co-created with the involvement of end-users and using their official social media accounts to disseminate such health-related promotional materials. In addition, governments and health authorities should empower factory workers with adequate skills to evaluate the veracity of information about booster doses of COVID-19 vaccine. 

## Figures and Tables

**Table 1:** Background characteristics of the participating factory workers who had completed their initial doses of COVID-19 vaccine and not received a booster dose (*n* = 2329).

	*n* (%)
**Sociodemographic**	
Age group, years	
18–29	614 (26.4)
30–39	1064 (45.7)
40–49	514 (22.1)
≥50	137 (5.9)
Gender	
Male	1147 (49.2)
Female	1182 (50.8)
Internal migrant	
No	265 (11.4)
Yes	2064 (88.6)
Relationship status	
Currently single	492 (21.1)
Having a stable boyfriend/girlfriend	164 (7.0)
Married	1673 (71.8)
Having children	
No	712 (30.6)
Yes	1617 (69.4)
Highest education level attained	
Junior high or below	580 (24.9)
Senior high or equivalent	678 (29.1)
College/university or above	1071 (46.0)
Monthly personal income, CNY (USD)	
<3000 (463.84)	332 (14.3)
3000–4999 (463.84–772.92)	648 (27.8)
5000–6999 (773.07, 1082.15)	766 (32.9)
7000–9999 (1082.30–1545.99)	386 (16.6)
≥10,000 (1546.14)	197 (8.5)
Type of work	
Frontline workers	1583 (68.0)
Management staff	746 (32.0)
Type of factory	
Electronic device manufacturers	1399 (60.1)
Other factories	930 (39.9)
**Uptake of COVID-19 vaccine**	
Time interval between the completion of initial doses and the survey date	
<1 month	40 (1.7)
1–3 months	872 (37.4)
4–6 months	1321 (56.7)
>6 months	96 (4.1)
Self-reported severity of side-effects after COVID-19 vaccination	
Not at all	1520 (65.3)
Very mild	500 (21.5)
Mild	276 (11.9)
Moderate	28 (1.2)
Severe	3 (0.1)
Very severe	2 (0.1)
Have not received any COVID-19 vaccine	0 (0.0)
**Personal COVID-19 preventive measures in the past month**	
Frequency of wearing a facemask in public places/transportation other than the workplace	
Every time	1996 (85.7)
Often	270 (11.6)
Sometimes	60 (2.6)
Never	3 (0.1)
Frequency of wearing a facemask when you have close contact with other people in the workplace	
Every time	1706 (73.3)
Often	470 (20.2)
Sometimes	137 (5.9)
Never	16 (0.7)
Self-reported sanitizing hands (using soaps, liquid soaps, or alcohol-based sanitizer) after returning from public spaces or touching public installation	
Every time	1284 (55.1)
Often	582 (25.0)
Sometimes	409 (17.6)
Never	54 (2.3)
Self-reported avoiding social gathering with other people who do not live together	
No	946 (40.6)
Yes	1383 (59.4)
Self-reported avoiding crowded places	
No	799 (34.3)
Yes	1530 (65.7)
**COVID-19 prevention measures implemented by the factories in the past month**, *n* (%) yes	
Prohibiting non-employees from entering workplace	1622 (69.6)
Taking body temperature and sanitizing hands for all employees entering the workplace	1940 (83.3)
Providing facemasks to all employees	1995 (85.7)
Keeping adequate distance (e.g., >1 m) between work stations	
Requiring employees to wear facemasks when they have close contact with other people	1725 (74.1)
Frequent workplace disinfection	1988 (85.4)
Maintaining adequate ventilation in workplace	1953 (83.9)
Setting up partitions in factory canteens	2089 (89.7)
Requiring employees to receive COVID-19 vaccination	1724 (74.0)
Number of COVID-19 prevention measures implemented by the factories, mean (SD)	7.2 (2.5)

**Table 2:** Perceptions related to booster dose of COVID-19 vaccine among factory workers who had completed initial doses of COVID-19 vaccine and not received a booster dose (*n* = 2329).

	*n* (%)
**Behavioral Intention**	
Intention to get a booster dose of COVID-19 vaccine in the next six months	
Very unlikely/unlikely/neutral	373 (16.0)
Likely/very likely	1956 (84.0)
**Individual-level factors**	
Perceived risk of contracting Delta variant of COVID-19, *n* (%) high	1397 (60.0)
Response score, mean (SD)	2.6 (0.5)
Perceived chance of having severe consequence if contracting Delta variant of COVID-19, *n* (%) high	1099 (47.2)
Response score, mean (SD)	2.4 (0.6)
Perceived benefit of booster dose of COVID-19 vaccine, *n* (%) agree	
Receiving a booster dose can maintain your antibody level and strengthen the protection against COVID-19	1786 (76.7)
A booster dose is highly effective in protecting you from COVID-19	1728 (74.2)
China has sufficient supply of booster doses of COVID-19 vaccines	1571 (67.5)
Perceived Benefit Scale ^1^	
Scale score, mean (SD)	8.1 (1.2)
Perceived barrier of receiving booster dose of COVID-19 vaccine, *n* (%) agree	
You will have severe side effects after receiving a booster dose	331 (14.2)
The harms of a booster dose is unclear in long run	383 (16.4)
The protection of a booster dose will only last for a short time	393 (16.9)
Perceived Barrier Scale ^2^	
Scale score, mean (SD)	5.7 (1.6)
People who are important to you will suggest you to receive a booster dose of COVID-19 vaccine, *n* (%) agree	1708 (73.3)
Response score, mean (SD)	2.7 (0.5)
It is easy for you to receive a booster dose of COVID-19 vaccine if you want to, *n* (%) agree	1454 (62.4)
Response score, mean (SD)	2.6 (0.6)
The SURE test version of Decisional Conflict Scale, *n* (%) Yes	
There are different choices of a booster dose…	
Do you feel sure about the best choice for you?	856 (36.8)
Do you know the benefits and risks of each option?	767 (32.9)
Are you clear about which benefits and risks matter most to you?	669 (28.7)
Do you have enough support and advice to make a choice?	918 (39.4)
The SURE test version of Decisional Conflict Scale ^3^	
Scale score, mean (SD)	1.4 (1.6)
**Interpersonal-level variables**	
Frequency of exposing to the following information on social media (e.g., WeChat, WeChat moments, Weibo, TikTok) in the past month	
COVID-19 pandemic is not under control in some countries after scaling up COVID-19 vaccination	
Almost none	464 (19.9)
Seldom	725 (31.1)
Sometimes	705 (30.3)
Always	435 (18.7)
Response score, mean (SD)	2.5 (1.0)
Infectiousness and harms of the Delta variant of COVID-19	
Almost none	299 (12.8)
Seldom	590 (25.3)
Sometimes	803 (34.5)
Always	637 (27.4)
Response score, mean (SD)	2.8 (1.0)
Outbreak of Delta variant of COVID-19 in some places of China	
Almost none	376 (16.1)
Seldom	782 (33.6)
Sometimes	820 (35.2)
Always	351 (15.1)
Response score, mean (SD)	2.5 (0.9)
People contract COVID-19 after receiving primary series of COVID-19 vaccination	
Almost none	522 (22.4)
Seldom	1048 (35.0)
Sometimes	622 (26.7)
Always	137 (5.9)
Response score, mean (SD)	2.1 (0.8)
Thoughtful consideration of the veracity of COVID-19-specific information	
Almost none	368 (15.8)
Seldom	523 (22.5)
Sometimes	790 (33.9)
Always	648 (27.8)
Response score, mean (SD)	2.7 (1.0)
Satisfaction of COVID-19 vaccination health promotion materials (e.g., advertisement, poster, and others) produced by the government	
Amount of information	
Just right	1695 (72.8)
Too much	431 (18.5)
Too little	203 (8.7)
Can address your concerns related to COVID-19 vaccination	
No/uncertain	795 (34.1)
Yes	1534 (65.9)
Helpful for you to make decision on whether to receive a COVID-19 vaccine	
No/uncertain	293 (12.6)
Yes	2036 (87.4)

^1^ Perceived Benefit Scale: three items, Cronbach’s alpha: 0.78; one factor was identified by exploratory factor analysis, explaining 70.5% of total variance. ^2^ Perceived Barrier Scale: three items, Cronbach’s alpha: 0.79; one factor was identified by exploratory factor analysis, explaining 70.1% of total variance. ^3^ The SURE test version of the Decisional Conflict Scale, Cronbach’s alpha: 0.88; one factor was identified by exploratory factor analysis, explaining 72.9% of total variance.

**Table 3:** Associations between background characteristics and behavioral intention to receive a booster dose of COVID-19 vaccine within the next six months.

	OR (95% CI)	*p* Values
**Sociodemographic**		
Age group, years		
18–29	1.0	
30–39	0.86 (0.66, 1.13)	0.27
40–49	1.04 (0.75, 1.45)	0.81
≥50	1.20 (0.70, 2.06)	0.52
Gender		
Male	1.0	
Female	1.26 (1.01, 1.58)	0.04
Internal migrant		
No	1.0	
Yes	1.18 (0.85, 1.65)	0.32
Relationship status		
Currently single	1.0	
Having a stable boyfriend/girlfriend	1.78 (1.05, 3.02)	0.03
Married	1.26 (0.97, 1.64)	0.08
Having children		
No	1.0	
Yes	1.23 (0.97, 1.55)	0.09
Highest education level attained		
Junior high or below	1.0	
Senior high or equivalent	1.26 (0.95, 1.68)	0.11
College/university or above	1.64 (1.25, 2.15)	<0.001
Monthly personal income, CNY (USD)		
<3000 (463.84)	1.0	
3000–4999 (463.84–772.92)	1.32 (0.94, 1.86)	0.11
5000–6999 (773.07, 1082.15)	1.59 (1.14, 2.23)	0.01
7000–9999 (1082.30–1545.99)	1.23 (0.84, 1.79)	0.29
≥10,000 (1546.14)	1.35 (0.85, 2.16)	0.20
Type of work		
Frontline workers	1.0	
Management staff	1.46 (1.14, 1.88)	0.003
Type of factory		
Electronic device manufacturers	1.0	
Other factories	1.03 (0.82, 1.29)	0.82
**Uptake of COVID-19 vaccine**		
Time interval between the completion of initial doses and the survey date		
≤3 month	1.0	
4–6 months	1.10 (0.87, 1.38)	0.43
>6 months	1.76 (0.90, 3.50)	0.10
Self-reported severity of side-effects after COVID-19 vaccination		
Not at all	1.0	
Very mild/mild	1.09 (0.86, 1.39)	0.47
Moderate/severe/very severe	0.52 (0.24, 1.13)	0.09
**Personal COVID-19 preventive measures in the past month**		
Frequency of wearing a facemask in public places/transportation other than the workplace		
Never/sometimes/often	1.0	
Every time	1.46 (1.09, 1.95)	0.01
Frequency of wearing a facemask when you have close contact with other people in the workplace		
Never/sometimes/often	1.0	
Every time	1.14 (0.89, 1.46)	0.29
Self-reported sanitizing hands (using soaps, liquid soaps, or alcohol-based sanitizer) after returning from public spaces or touching public installation		
Never/sometimes/often	1.0	
Every time	0.96 (0.77, 1.20)	0.70
Self-reported avoiding social gathering with other people who do not live together		
No	1.0	
Yes	1.55 (1.24, 1.93)	<0.001
Self-reported avoiding crowded places		
No	1.0	
Yes	1.61 (1.29, 2.02)	<0.001
**COVID-19 prevention measures implemented by the factories in the past month**		
Number of COVID-19 prevention measures implemented by the factories	1.12 (1.08, 1.17)	<0.001

OR: crude odds ratios. CI: confidence interval.

**Table 4:** Factors associated with behavioral intention to receive a booster dose of COVID-19 vaccine within the next six months.

	OR (95% CI)	*p* Values	AOR (95% CI)	*p* Values
**Individual-Level Factors**				
Perceived risk of contracting Delta variant of COVID-19	1.67 (1.36, 2.05)	<0.001	1.53 (1.24, 1.90)	<0.001
Perceived chance of having severe consequence if contracting Delta variant of COVID-19	1.59 (1.31, 1.93)	<0.001	1.45 (1.18, 1.77)	<0.001
Perceived Benefit Scale	1.73 (1.59, 1.88)	<0.001	1.71 (1.57, 1.87)	<0.001
Perceived Barrier Scale	0.80 (0.74, 0.85)	<0.001	0.80 (0.74, 0.85)	<0.001
People who are important to you will suggest you to receive a booster dose of COVID-19 vaccine	3.22 (2.67, 3.89)	<0.001	2.53 (2.09, 3.77)	<0.001
It is easy for you to receive a booster dose of COVID-19 vaccine if you want to	2.20 (1.86, 2.60)	<0.001	2.04 (1.72, 2.43)	<0.001
The SURE test version of Decisional Conflict Scale	1.12 (1.04, 1.20)	0.003	1.07 (0.99, 1.15)	0.10
**Interpersonal-level variables**				
Frequency of exposing to the following information on social media (e.g., WeChat, WeChat moments, Weibo, TikTok) in the past month				
COVID-19 pandemic is not under control in some countries after scaling up COVID-19 vaccination	1.26 (1.12, 1.40)	<0.001	1.26 (1.13, 1.41)	<0.001
Infectiousness and harms of the Delta variant of COVID-19	1.40 (1.25, 1.56)	<0.001	1.38 (1.23, 1.54)	<0.001
Outbreak of Delta variant of COVID-19 in some places of China	1.31 (1.16, 1.48)	<0.001	1.32 (1.16, 1.49)	<0.001
People contract COVID-19 after receiving primary series of COVID-19 vaccination	1.14 (1.00, 1.31)	0.051	1.13 (0.99, 1.30)	0.08
Thoughtful consideration of the veracity of COVID-19-specific information	1.38 (1.24, 1.54)	<0.001	1.35 (1.21, 1.50)	<0.001
Acceptance of COVID-19 vaccination health promotion materials (e.g., advertisement, poster, and others) produced by the government				
Amount of information				
Just right	1.0		1.0	
Too much	0.79 (0.60, 1.05)	0.10	0.77 (0.58, 1.03)	0.08
Too little	0.57 (0.40, 0.81)	0.002	0.70 (0.48, 1.00)	0.052
Can address your concerns related to COVID-19 vaccination				
No/uncertain	1.0		1.0	
Yes	2.32 (1.85, 2.90)	<0.001	2.04 (1.62, 2.57)	<0.001
Helpful for you to make decision on whether to receive a COVID-19 vaccine				
No/uncertain	1.0		1.0	
Yes	4.45 (3.40, 5.82)	<0.001	3.87 (2.91, 5.14)	<0.001

OR: crude odds ratios CI: confidence interval AOR: adjusted odds ratios, odds ratios adjusted for significant background characteristics (gender, relationship status, highest education level attained, monthly personal income, type of work, frequency of wearing a facemask in public spaces/transportation other than the workplace, self-reported avoidance of social gatherings and crowded places, and number of COVID-19 prevention measures implemented by their factory).

## Data Availability

The data presented in this study are available from the corresponding author upon request. The data are not publicly available as they contain personal behaviors.
